# Case report: Recurrent parosteal lipoma at Dr. Moewardi hospital

**DOI:** 10.1016/j.amsu.2022.104061

**Published:** 2022-07-05

**Authors:** Widyanti Soewoto, Brian Waskita, Amriansyah Pranowo Imamsoedjana

**Affiliations:** a Surgical Oncology Subdivision, Dr. Moewardi Hospital, Faculty of Medicine, Sebelas Maret University, Jalan Slamet Riyadi 419 Makamhaji Kartasura Sukoharjo Jawa Tengah Indonesia, 57161, Indonesia; b Pathology Anatomy Department, Dr. Moewardi Hospital, Faculty of Medicine, Sebelas Maret University, Bagian Patologi Anatomi RS Dr Moewardi Surakarta. Jalan Kol. Sutarto 132 Jebres Surakarta Jawa Tengah Indonesia, 57126, Indonesia; c Department of Surgery, Faculty of Medicine, Sebelas Maret University, Bagian Bedah RS Dr Moewardi Surakarta. Jalan Kol. Sutarto 132 Jebres Surakarta Jawa Tengah Indonesia, 57126, Indonesia

**Keywords:** *Case report*, *Rare cases*, *Parosteal lipoma*, *Relapsed*

## Abstract

**Background:**

Parosteal lipoma is a rare and benign neoplasm originating from mature adipose tissue near the periosteum. Clinically, it is difficult to diagnose due to its similarity to the clinical manifestation of sarcoma, so imaging, histopathology, and immunohistochemistry examinations are necessary.

**Case presentation:**

A 54-year-old woman presented with lump on the right thigh that had gone through surgery eight years prior, with a diameter of 20 cm, with a partly hard and partly soft consistency, the patient was diagnosed with suspected recurrent liposarcoma. We performed wide excision and histopathological results showed a proliferation of bone cells and cartilage cells that were lobulated, surrounded by a proliferation of fat cells with no pleomorphism or immature cells.

**Discussion:**

Parosteal lipomas are neoplasms derived from adult adipose tissue, usually connected to the periosteum, and rare and benign. Two theories of pathogenesis of parosteal lipomas. (1) tumors arise from the differentiation of stem cells derived from adipose tissue, (2) the tumor is derived from secondary metaplasia of fibroblasts due to recurrent trauma, metabolic changes, or ischemia. Based on the theory, it is likely that in this case is due to the presence of differentiation of adiposa tissue due to the non-acquisition of a history of trauma.

**Conclusion:**

Parosteal lipoma is a rare case of benign neoplasm, which is difficult to diagnose clinically due to its similar sarcoma, thus requiring imaging and histopathological examination. The treatment of choice is wide excision by taking the entire tumor to prevent a recurrence.

## Highlights

• 45 year old woman with recurrent parosteal lipoma.
• The case of parosteal lipoma is a benign tumor.
• A neoplasm originating from mature adipose tissue, mostly located in the bone cortex below the periosteum.
• The incidence is 0.3% of all types of lipomas and 0.1% of primary bone neoplasms.

## 1 Introduction

Parosteal lipoma is a rare subtype of lipoma located close to the periosteum and takes the form of a bone protrusion. Microscopically, parosteal lipoma appears as bone and cartilage against a background of predominantly mature adipose tissue [1]. Clinically, a parosteal lipoma is difficult to diagnose. For that reason, it requires further examination using imaging modalities such as X-ray, CT scan, or MRI and is confirmed by pathological results [2]. The following case reports a woman with a lump on her right thigh clinically diagnosed with recurrent liposarcoma with a previous history of surgery of a lipoma and following the SCARE criteria [3].

## 2 Presentation of case

A 54-year-old housewife with a lump on the right thigh. The patient was referred from a peripheral hospital, with a history of eight years ago there was a lump in the same place as the diameter 5 cm, a surgical removal was performed by a general surgeon with the results of the pathological anatomy of the lipoma. The patient reported having two lumps with a diameter of 20 cm; The first lump is located on the lateral side with a soft consistency, while the second lump is located on the medial side with a hard consistency. The patient does not feel pain or burning sensation in both lumps. There is no history of trauma, and there is no family history with cancer.

The results of MSCT with contrast showed solid and some semi-solid lesions, which consisted of calcifications and fat at the one-third proximal to one-third distal of the femur. The lesions had smooth borders with partly irregular and septated edges with the size of 23 × 16 × 27 cm.

At Dr Moewardi Hospital, our surgery is performed in a team, an oncology surgeon as an operator with anesthesia performed regionally. The patient's position is tilted to the left, the lump is located above. We perform a wide excision, obtaining an intermuscular encapsulation mass with a soft consistency in the lateral position and a hard surface on the medial part resembling a bone. All lumps were successfully removed. Post surgery is not found interference in carrying out activities.

The microscopic examination showed the proliferation of bone cells and cartilage cells, which were lobulated, surrounded by proliferating fat cells. There were no signs of malignancy. As for the immunohistochemical examination, S-100, vimentin, SMA, and HMB-45 showed positive results.

## 3 Discussion

Dr. Seerig first discovered parosteal lipoma in 1836. It is a neoplasm originating from mature adipose tissue, usually connected to the periosteum, mostly located in the bone cortex below the periosteum, and is rare and benign. The incidence is 0.3% of all types of lipomas and 0.1% of primary bone neoplasms. The term parosteal lipoma was first made famous by D'Arcy Power in 1888, which implies the relationship between the tumor and the bone while not always originating from the bone (periosteum) [4]. Parosteal lipoma is the preferred term for periosteal lipoma because the periosteal tissue does not contain fat cells. The most common predilection sites are the femur, proximal part of the radius, humerus, tibia, clavicle, and pelvis. The ribs can be affected, but it is quite rare [5,6].

Parosteal lipoma consisting of fat tissues and some differentiation of bones and cartilage is usually located inside the subcutis fat or in the muscles [2,7]. Therefore, patients’ complaints also vary depending on the site of occurrence. Parosteal lipoma can be diagnosed through history, physical examination, and further workup. Patients with parosteal lipoma usually complain of a painless mass with no motoric or sensory dysfunction. Lymph nodes examination also shows no lesions. In several cases, parosteal lipoma can be difficult to diagnose clinically [8].

Debras et al. reported a patient with a parosteal lipoma on the leg. Physical examination showed a painless, mobile, and slowly growing mass with a hard consistency, with a firm border, of similar color to the skin or the surrounding mucosa, without any signs of inflammation, no signs of limitations in the range of motion (ROM) [2,8,9,10].

In our case, the lump was found on the femur. The lump made an impression of two separate masses, in which the lateral lobe had a more superficial tumor with a soft consistency. In contrast, the lump at the medial was located deeper and closer to the femur and was hard inconsistency. With no ulceration, tenderness, swelling, erythema, or another skin lesion, the mass slowly grew. The most prominent finding of this case was the history of surgery at the exact location 8 years prior. The history of surgery at the same site and the hard consistency of the lump were the reasons why the lump was initially clinically diagnosed as recurrent mixed liposarcoma.

Parosteal lipomas are difficult to distinguish from parosteal sarcoma, osteochondroma, liposarcoma, lipoma, and chondrosarcoma, clinically may appear with similar clinical manifestations. In our case, clinically the lump is more similar as a mixed liposarcoma. based on its prevalence, recurrency of parosteal lipoma does not occur after intoto-excision of the tumor, while for liposarcoma, there is still a 33.8% chance of recurrence [11].

Based on the classification of soft tissue tumors by WHO 2002, conventional lipoma consists of mature adipose tissue. It is located in the subcutaneous region (superficial lipoma) or inside the soft tissue (lipoma), or even on the surface of the bone (parosteal lipoma). Intraosseous lipoma is located inside the bone. The terms osteolipoma and ossifying lipoma are currently used for soft tissue tumors with no relation to the periosteum. In contrast, parosteal lipoma is used for tumors with relation to the periosteum [12].

The microscopic examination found partly encapsulated lesions mainly consisting of lobules of univaculated mature adipocytes and bone trabeculae, with evenly spread lands of benign cartilage. Between the adipose tissue, proliferation of fibroblast, chondrocyte, osteocyte, and blood vessels with thick walls could be seen. Osteocytes and osteoclasts surrounded the bone trabeculae. Mixed liposarcoma is a malignant tumor consisting of circle to oval-shaped primitive mesenchymal cells and a variable number of small signet ring lipoblasts in the myxoid stroma. Liposarcoma differentiates well and is also known as an atypical lipomatous tumor. It is a locally aggressive and malignant mesenchymal neoplasm composed of mature adipocytic proliferation, which exhibits significant malignant cytology; it may occasionally show areas of chondroid metaplasia without extensive myxochondroid matrix [13]. In this case, neither pleomorphism nor immature cell features were seen. Thus, the diagnosis of mixed mesenchymal liposarcoma could be ruled out.

Immunohistochemical staining of liposarcomas showed that the more mature the adipoblast differentiation, the higher the positive level of vimentin and S-100 expression. Moreover, the nuclear proliferation index was <1%, and the Ki-67 proliferation index was low. KP1, LAM, CD68, and CK showed positive results in some cases, while EMA, SMA, MSA, HMB45, and GFAP would give negative results [14]. In this patient, S-100, vimentin, SMA, HMB-45, and EMA showed positive results.

There are two main theories for the pathogenesis of parosteal lipoma. (1) These tumors may arise from multidirectional differentiation of stem cells derived from adipose tissue, or (2) the tumors originate from secondary metaplasia of fibroblasts due to repeated trauma, metabolic changes, or ischemia. Several cytogenetic studies on osteolipomas and parosteal lipomas have shown similarities to the t(3; 12) translocation in conventional soft tissue lipoma. By identifying the HMGA2-LPP fusion transcription in both the fibro-adipose component and the chondro-osseous component of tendon lipomas, it revealed that the HMGA2-LPP fusion protein induces fibroadipogenesis and osteochondrogenesis [12].

Lipomas found in adults often consist of chromosome 12q13∼15 aberration, which causes rearrangement in the HMGA2 gene at the 12q14.3, with breakpoints occurring inside or outside the gene. Panagopoulos studied eleven lipomas and one osteochondrolipoma with a new recurrent chromosomal aberration, t(12; 18) (q14∼15; q12∼21). The molecular study conducted on eight tumors showed three cases expressing full-length HMGA2 transcripts and five cases with chimeric HMGA2 transcripts. Of the three lipomas and osteochondrolipoma, exons 1–3 of the HMGA2 was coupled to the SETBP1 sequence at 18q12.3 or the intragenic sequence of 18q12.3, about 10 kbp distal to SETBP1. In other lipomas, exons 1–4 of HMGA2 fuse to the intronic sequence GRIP1, which maps to the band chromosome 12q14.3, distal to HMGA2. Subsequent HMGA2 fusion would code for the transcript of a putative protein suspected of containing an HMGA2 amino acid residue corresponding to exons 1–3 (or exons 1–4 in one case) followed by an amino acid residue corresponding to the fused sequence.

Further workup for parosteal lipoma includes:

a) X-ray and Computed Tomography scan (CT scan)

According to Miller 1992, plain X-ray examination of parosteal lipoma in long bones could be classified into 4 types. **Type I.** Lucent lesion with dense fat directly adjacent to the radial cortex with no relations to bone changes. **Type II.** A lenticular mass lesion with a smooth surface of fat density bordering the long midshaft segment. **Type III.** The AP radiograph depicts a low protrusion of elevation of the medial femoral diaphysis with irregular bone density mixed with areas of fat density. **Type IV.** Direct imaging shows irregular protrusion of bone density in a density of oval fat.

CT scan plays a crucial role in evaluating parosteal lipoma and is useful for documenting the presence of fat and bony elements. In demonstrating the presence of bony elements and evaluating the relationship of the mass to the surrounding bone structures (erosion and/or continuity), a CT scan is the procedure of choice. 3D reconstruction does not add much-needed information, but it may help localize the tumor and demonstrate the anatomical orientation of the mass, a parameter that facilitates excision. In addition, a CT scan provides a fine picture of scattered calcifications. On X-ray and CT scans, parosteal lipoma appears as a calcified soft tissue mass [2,8,9,10].

b) Magnetic Resonance Imaging (MRI)

Although CT scan is useful in visualizing fat and bony elements, MRI is considered the imaging modality for lipomatous tumors. MRI allows the identification of lipomas, which has a homogenously identical signal intensity to that of subcutaneous fat in all pulse sequences, whereas ossification, calcification, and fibrous connective tissue appear as low signal intensity [2,9].

**Fig. 1:**
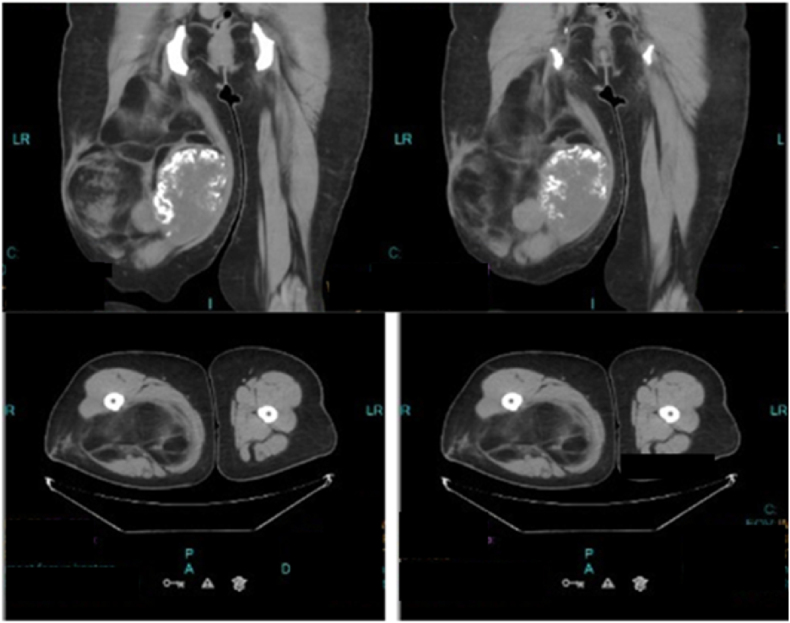
MSCT with contrast 15–46 HU, one-third proximal – one-third distal of the femur region.

The imaging findings show chondroid lipoma, fatty tumors with chondro-osseous differentiation such as lipomas or liposarcoma, tumors including a myxoid component such as myxoid liposarcoma and extraskeletal myxoid chondrosarcoma, should also be included in the differential diagnosis. Both lesions may show heterogeneous signal changes on MRI associated with varying amounts of fat and chondroid tissue. A small number of a reticulated or linear pattern of mature fat and a cyst-like appearance found in myxoid liposarcomas can help differentiate them from chondroid lipoma. Its invasive nature can also help differentiate extraskeletal myxoid chondrosarcoma from chondroid lipoma [13]. Due to limitations in the insurance, an MRI examination was not performed on this patient. The results of the MSCT showed a typical picture of parosteal lipoma with the presence of fat and bone components (see Fig. 1).

**Fig. 2:**
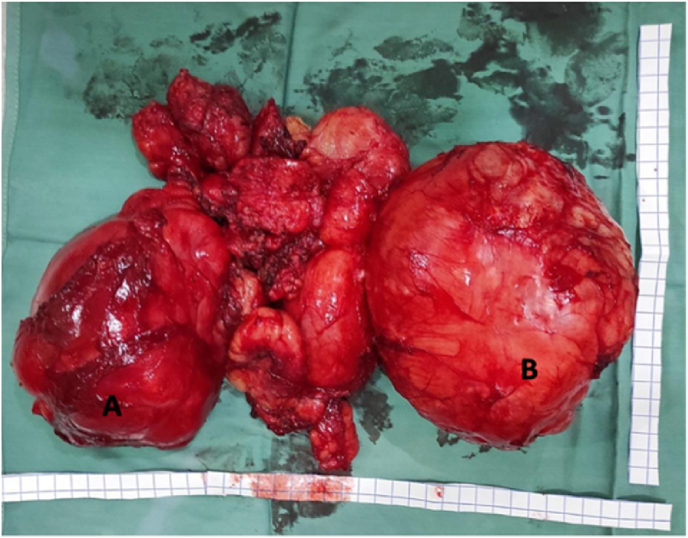
Macroscopic picture of a tumor divided into two encapsulated parts located intramuscularly. **A.** Hard mass suggesting an osteochondroma and **B**. Soft mass suggesting a lipoma.

**Fig. 3:**
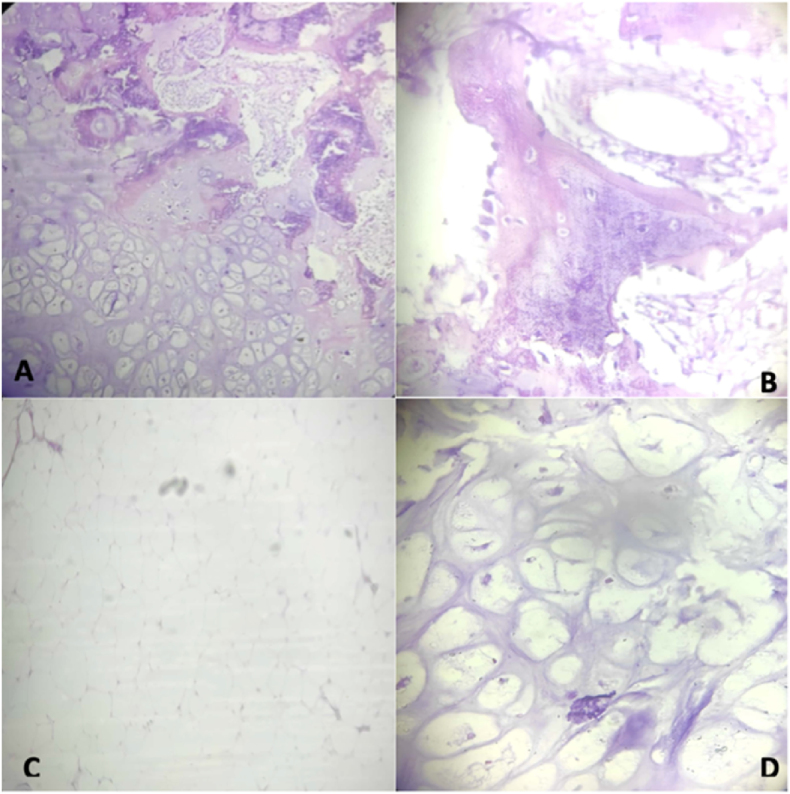
Histopathology **A.** Components of chondroid, osteoid matrix, and lipoma with a 40× magnification. **B** Components of osteoid **C.** Components of lipoma with a 100× magnification. **D** Components of chondroid with a 400× magnification.

**Fig. 4:**
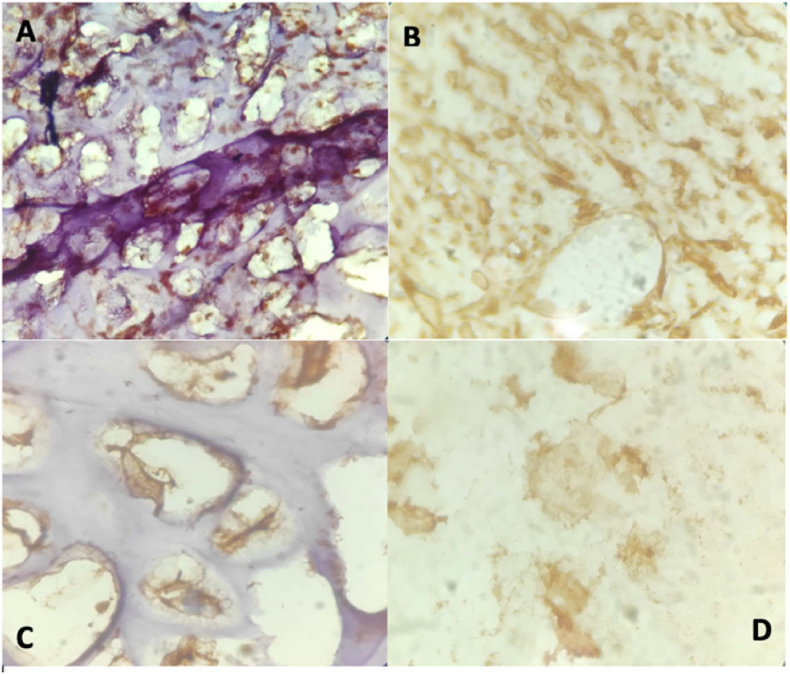
Immunohistochemistry. **A.** SMA with a magnification of 40. **B.** Vimentin with a magnification of 100. **C.** S100 with a magnification of 100. **D.** HMB-45 with a magnification of 100.

The treatment of choice for parosteal lipomas is excision of the entire tumor [15,7]. During the operation of this patient, the lobed lump (which is located superficially) looks like a lipoma, while the lump located close to the bone has the impression of bone tissue. Judging by these findings, the first operation 8 years earlier, took only superficial tissue in the form of pure adipose tissue compatible with the histological results of lipomas (Fig. 2. B), recurrence occurs because the tumor mass near the bone is not lifted. Based on the theory, it is likely that in this case parosteal lipoma is due to the presence of differentiation of adiposa tissue due to the non-acquisition of a history of trauma (see Fig. 4) (see Fig. 3).

The prognosis for parosteal lipomas is the same as for other lipomas, and the tumor usually does not recur after complete excision [2]. Evaluation for 6 months, the patient no abnormalities were obtained either clinically or imaginarily and there were no disturbances in daily activities.

## 4 Conclusion

Parosteal lipoma is a rare and benign case of neoplasm. It is difficult to diagnose clinically due to its similarities to the clinical manifestations of mixed liposarcoma, so imaging and histopathological examinations are needed. The treatment of choice for this tumor is wide excision, removing the entire tumor in toto to prevent a recurrence.

## Ethical approval

The patient gave consent to the author for publication.

## Sources of funding

The authors received no financial support for this article's research, authorship, and publication.

## Consent

Informed Consent in writing is obtained from the patient for the publication of the report of this case and the accompanying images.

## Author contribution

Widyanti Soewoto M.D., Ph.D.: study concept, data analysis and writing the paper. Brian Waskita M.D., Ph.D.: interpretation of anatomical pathology. Amriansyah Pranowo Imamsoedjana: data analysis.

## Registration of research studies

1. Name of the registry: Research registry.

2. Unique Identifying number or registration ID: researchregistry8022.

3. Hyperlink to your specific registration (must be publicly accessible and will be checked):

## Guarantor

Widyanti Soewoto M.D., Ph.D.

## Provenance and peer review

Not Commissioned, Externally Peer-Reviewed.

## Declaration of competing interest

The authors declare no potential conflicts of interest.
